# Catching the Storm Before It Breaks: Advancing Early Diagnosis and Intervention in Bipolar Disorder

**DOI:** 10.3390/life16010007

**Published:** 2025-12-20

**Authors:** Marianna Mazza, Luca Chisari, Francesco Maria Lisci, Caterina Brisi, Giovanni Camardese, Domenico De Berardis, Luigi Janiri, Gabriele Sani, Giuseppe Marano

**Affiliations:** 1 Department of Neuroscience, Head-Neck and Chest, Section of Psychiatry, Fondazione Policlinico Universitario Agostino Gemelli IRCCS, Largo Agostino Gemelli 8, 00168 Rome, Italy; lucachisari@yahoo.com (L.C.); fmlisci@gmail.com (F.M.L.); caterinabrisi8@gmail.com (C.B.); g.camardese@unilink.it (G.C.); luigi_janiri@fastwebnet.it (L.J.); gabriele.sani@unicatt.it (G.S.); 2 Department of Neuroscience, Section of Psychiatry, Università Cattolica del Sacro Cuore, 00168 Rome, Italy; 3 Department of Life Science, Health, and Health Professions, Link Campus University, 00165 Rome, Italy; 4 Department of Mental Health, ASL 4 Teramo, 64100 Teramo, Italy; domenico.deberardis@aslteramo.it

**Keywords:** bipolar disorder, early intervention, clinical staging, prevention, biomarkers, personalized psychiatry

## Abstract

In recent years, growing attention has been directed toward the early diagnosis and intervention of bipolar disorder (BD), driven by the recognition that timely clinical action may significantly alter the trajectory of the illness. Early identification of individuals at risk, as well as intervention during prodromal or subthreshold phases, offers the potential to delay onset, reduce episode severity, improve long-term outcomes, and possibly prevent the full manifestation of the disorder. This narrative review aims to provide a comprehensive overview of the current literature on the early stages of BD, including clinical high-risk states, neurobiological and cognitive markers, and psychosocial indicators. It also explores the latest research findings and their implications for clinical practice, highlighting the importance of integrated approaches that combine biomarker discovery, risk stratification models, and youth-focused mental health services. Finally, the review discusses the ethical and practical challenges of early intervention and underscores the need for further longitudinal studies and personalized preventive strategies.

## 1. Introduction

Bipolar disorder (BD) is a chronic, relapsing, and potentially progressive psychiatric condition that affects mood regulation, leading to episodes of mania, hypomania, and depression [1,2,3]. It often begins in adolescence or early adulthood and is associated with considerable personal, social, and economic burden. Traditionally, clinical management has focused on treating acute episodes and preventing relapses. However, there is a growing recognition that earlier identification and intervention could significantly improve outcomes. This has led to increasing attention toward prevention strategies, particularly those aimed at early detection and early intervention [4,5]. These approaches may delay illness onset, reduce symptom severity, slow progression, and in some cases, prevent full-blown BD. Early identification carries ethical challenges, including risk of overdiagnosis, stigma, false positives, and treatment decisions under uncertainty.

The evolving view of BD as a disorder with a reasonably predictable trajectory has significantly contributed to the conceptual foundation for early intervention. Staging models, which conceptualize BD as progressing through identifiable phases, have provided a useful framework for research and clinical care. These models are based on frameworks such as that proposed by Berk et al. [6] and further refined by Kapczinski et al. [7] and Scott et al. [8], which outline a progression from an asymptomatic at-risk phase (Stage 0), to subthreshold symptoms (Stage 1), first episode (Stage 2), recurrent episodes (Stage 3), and chronicity with functional decline (Stage 4). Staging models have been operationalized in empirical studies through prospective cohorts that classify individuals into at-risk, subthreshold, and first-episode stages based on clinical presentation, functional impairment, and biomarkers.

Following these models, BD begins with an “at-risk” period, followed by subthreshold symptomatology, the first mood episode, recurrent episodes, and eventually a chronic, treatment-resistant phase characterized by enduring functional decline. A substantial body of evidence supports the idea that for a significant proportion of patients, BD follows a progressive course. This progression is reflected not only in worsening clinical features, such as increased episode frequency and duration, persistent mood instability, and impaired functioning, but also in neurobiological alterations, including structural brain changes indicative of cumulative neurotoxic effects and decreased neurotrophic support [2,3,4,5,6,7,8,9]. Large-scale epidemiological surveys, such as the National Comorbidity Survey Replication (NCS-R), report that the median age of onset for BD is around 13 years, with nearly 75% of cases beginning before age 25 [10,11]. This reinforces adolescence and early adulthood as critical risk periods.

According to this framework, the early stages of BD present a critical window of opportunity for intervention. Timely treatment during the at-risk or prodromal phases may prevent or mitigate the neuroprogressive course of the illness, ultimately preserving cognitive functioning and psychosocial well-being. Delaying intervention until the illness becomes fully manifest may allow neurobiological damage and functional deterioration to become entrenched, resulting in poorer long-term outcomes and a more refractory course that requires more intensive and costly treatment strategies [2].

A constellation of prodromal symptoms frequently precedes the onset of the first manic or hypomanic episode. Among the most commonly reported are mood lability, cyclothymic temperament, subclinical depressive symptoms, irritability, racing thoughts, and increased psychomotor activity [4,12]. These symptoms, although often nonspecific and subtle, may begin to emerge during adolescence or even earlier, influencing the individual’s emotional development and social functioning. Since the peak onset period for BD typically falls in late adolescence and early adulthood, a better understanding of these early clinical signals is essential for developing risk stratification tools and tailored early intervention protocols [13,14].

In this context, the early stages of BD represent both a challenge and an opportunity for modern psychiatry. On one hand, early symptoms may overlap with normative developmental experiences or other psychiatric conditions, making accurate identification difficult. On the other hand, the ability to detect and treat BD in its earliest stages could represent a paradigm shift in how the disorder is managed, shifting from reactive to proactive care, with the potential for far-reaching benefits in terms of quality of life, functional outcomes, and healthcare costs. A review by Keramatian et al. [15] outlines how early symptom profiles and family history can be combined with digital markers to enhance prediction accuracy in youth at clinical high risk for BD. Longitudinal cohorts consistently show that delayed diagnosis is associated with higher rates of relapse, functional decline, suicide attempts, and treatment resistance. Early intervention, particularly in youth at familial risk, has been linked to reduced conversion rates, improved psychosocial functioning, and attenuated symptom progression [2,12].

The objective of this narrative review is to synthesize the available literature on early diagnosis and intervention in BD, highlight neurobiological and psychosocial risk markers, evaluate the evidence supporting clinical staging models, and outline future directions toward personalized preventive psychiatry.

Compared with previous works on early stages of BD, this review offers an integrated framework that simultaneously synthesizes clinical prodromes, developmental trajectories, neurobiological correlates, and psychosocial determinants. Existing works tend to focus on isolated components, such as staging models, neuroimaging, or youth at familial risk, whereas our contribution lies in proposing a cross-domain synthesis aimed at operationalizing early identification in everyday clinical practice. This review argues that a multidimensional, developmentally informed, and ethically grounded approach is essential to advance early diagnosis and intervention in BD.

## 2. Materials and Methods

This paper is a narrative literature review aimed at synthesizing current knowledge on early diagnosis and early intervention in BD. Given the exploratory and integrative nature of the review, a non-systematic search strategy was employed to identify relevant publications from the contemporary literature. A literature search was conducted primarily through the PubMed/MEDLINE database. The search strategy included combinations of the following terms: bipolar disorder, early detection, early intervention, clinical high risk, prodrome, bipolar at risk, first episode mania, staging models, and biomarkers. Additional Medical Subject Headings (MeSH) terms were used when appropriate to broaden the scope. The search was limited to peer-reviewed articles published in English within the last two decades, although seminal earlier works were also included when clinically or conceptually relevant. Reference lists of key reviews and primary studies were examined to identify additional pertinent literature. No formal criteria for selection, exclusion, or methodological appraisal of studies were applied, as the objective was to provide a broad, integrative overview rather than a systematic synthesis of evidence. The initial search yielded 152 articles; after title/abstract screening, 81 were considered for full-text review, and 44 were included based on conceptual relevance. We selected PubMed because our focus was on clinical and neurobiological literature; nevertheless, we complemented the search with manual inspection of key references in developmental and psychosocial domains to mitigate database limitations. Articles were included if they contributed conceptually to understanding early stages of BD, defined as providing empirical evidence or theoretical models relevant to prodromal symptoms, staging, biomarkers, or early interventions. To minimize selection bias, reference lists of key papers were cross-checked, and seminal negative findings were explicitly considered. When evidence was contradictory, we compared methodological features (sample size, design, age range) and highlighted the degree of consensus or uncertainty accordingly.

## 3. Clinical Presentation of BD in Early Stages

The clinical presentation of BD in its early stages is complex, heterogeneous, and influenced by a variety of individual, genetic, and environmental factors [1]. One of the main challenges in early identification lies in the broad variability in symptom type, severity, and evolution over time. Early manifestations can range from subtle affective disturbances to more clearly defined mood episodes, and their clinical interpretation often requires differentiation between normative emotional fluctuations and pathological processes [2].

A crucial distinction in early presentation involves differentiating between the full-blown manic episodes characteristic of bipolar I disorder (BD-I) and the more subtle hypomanic features associated with bipolar II disorder (BD-II). While BD-I may present with an abrupt onset of overt manic symptoms, such as elevated mood, grandiosity, and psychotic features, BD-II typically unfolds with more nuanced hypomanic episodes that are often underrecognized or misattributed to personality traits or external stressors. Despite their differences in intensity and visibility, both manic and hypomanic symptoms may be preceded by a prodromal phase, which represents a valuable window for early detection and intervention [4,5].

Clinical observations and longitudinal studies [12,13] support the existence of a latency or prodromal period preceding the first major mood episode. This phase may last months or even years and is often marked by subthreshold symptoms that are not yet sufficient for a formal diagnosis [13]. Recognizing this early phase—particularly in high-risk individuals—could be key to altering the natural course of the illness. Identifying patterns of prodromal symptoms and understanding their temporal dynamics may enable clinicians to implement preventive strategies and potentially delay or mitigate the onset of full-threshold BD.

Recent frameworks emphasize stage-specific strategies that incorporate biomarkers, psychosocial factors, and clinical trajectories to inform early personalized interventions [16]. BD is also among the most highly heritable psychiatric disorders, with genetic factors contributing significantly to the risk, estimated to be between 60% and 85% of the variance [17,18]. Accordingly, a positive family history remains the strongest known predictor for the development of illness. In individuals with a genetic predisposition, prodromal symptoms may be more pronounced and more predictive of transition to syndromal BD. Genetic susceptibility plays a central role in early risk for BD. Large genome-wide association studies consistently implicate genes such as CACNA1C, ANK3, ODZ4, and loci linked to calcium-channel regulation, synaptic plasticity, and neuronal signaling. Although these variants confer small individual effects, polygenic risk scores significantly increase predictive accuracy when combined with clinical and familial data, supporting their potential role in multimodal early risk stratification [17].

Retrospective and prospective studies have consistently reported a range of putative prodromal symptoms, including mood lability, cyclothymic temperament, subclinical depressive episodes, racing thoughts, irritability, anxiety, and increased psychomotor activity [4,19]. These symptoms, although often nonspecific, tend to appear during adolescence or early adulthood, coinciding with a critical period of brain maturation and psychosocial development [13,14]. The challenge lies in distinguishing these early signs of BD from the transient emotional instability typical of adolescence.

Interestingly, some retrospective data suggests that individuals who later develop BD may report behavioral and emotional symptoms as early as toddlerhood, with signs such as excessive crying, sleep disturbances, and heightened sensitivity [20,21]. While these early manifestations are nonspecific and common in the general population, they may indicate early neurodevelopmental vulnerability, especially when seen in combination with family history and persistent mood dysregulation.

Emotional abuse and neglect, particularly when experienced during sensitive developmental periods, have long-lasting effects on the individual’s affect regulation systems, attachment style, and cognitive-emotional processing. From a psychodynamic perspective, early relational trauma disrupts the development of stable internal representations of self and others, contributing to chronic feelings of unworthiness, helplessness, and abandonment fears [22]. These internal schemas increase the vulnerability to interpersonal stressors and amplify the likelihood of depressive responses to relatively minor emotional challenges during remission.

Neurocognitive studies further demonstrate that individuals with a history of emotional maltreatment exhibit hyperactivation of limbic regions (e.g., amygdala) and altered connectivity in prefrontal areas involved in emotion regulation and cognitive control [23]. This dysregulation persists into adulthood and may reduce the capacity to buffer emotional distress, thereby predisposing to relapse. Additionally, deficits in mentalization and reflective functioning, frequently observed in maltreated individuals, impair the ability to make sense of one’s own emotional states and those of others [24], which in turn hinders effective coping in interpersonal contexts.

Cognitively, emotional abuse is linked to the development of negative attributional styles, hopelessness, and dysfunctional core beliefs [25,26], all of which are established predictors of depressive relapse. Finally, recent models emphasize the role of emotional neglect in shaping alexithymic traits and affective dysregulation [25,26,27], suggesting that emotional under-engagement may be just as detrimental as overt abuse in perpetuating depressive cycles. In summary, the early clinical presentation of BD is characterized by a constellation of variable and often ambiguous symptoms. However, by integrating familial, developmental, and symptomatic data, it may be possible to identify individuals at high risk and intervene during the early stages, potentially modifying the course of the illness before the onset of major mood episodes. Notably, studies such as Cannon et al. [28] and Tandon et al. [29] have reported positive predictive values (PPVs) ranging from 30% to over 50% when prodromal symptoms co-occur with familial risk. (Table 1).

Beyond clinical observation, understanding the neurobiological underpinnings of early-stage BD is crucial to identifying objective risk markers and informing targeted intervention.

Although numerous symptoms have been described in the early phases of BD, high-quality longitudinal evidence indicates that only a subset demonstrates consistent predictive validity. Across prospective offspring studies, youth with a first-degree family history who develop BD typically present with subthreshold hypomanic symptoms, episodic mood lability, cyclothymic or irritable temperament, and sleep–wake dysregulation several years before syndromal onset. Among these, subthreshold hypomania, affective instability, and psychotic-like experiences show the strongest associations with transition to BD, particularly when they co-occur with familial loading. In contrast, nonspecific symptoms such as anxiety, inattention, or general mood disturbances are prevalent but less predictive when considered in isolation.

To enhance clinical utility, Table 2 summarizes the prodromal markers with the highest predictive value, the quality of supporting evidence, and their observed effect direction. The available evidence is classified into high-, moderate-, and low-quality categories based on methodological rigor and consistency across studies.

## 4. Summary of Current Evidence on Early Stages of BD

The current literature on early diagnosis and intervention in BD is rich and diverse, encompassing various types of study designs, each offering unique insights into the early stages and prodromal development of the disorder. These include offspring studies, retrospective analyses, and prospective longitudinal studies focusing either on the first episode of mania or on individuals at clinical or familial risk for developing BD.

Offspring studies have been instrumental in clarifying the genetic underpinnings of BD. These studies investigate children of parents with BD and provide valuable data on how genetic vulnerability interacts with environmental and developmental factors [43,44]. They have consistently shown that children with a first-degree relative affected by BD are at significantly higher risk of developing mood disorders themselves. These findings underscore the importance of familial history as a powerful predictive factor and offer a window into the neurodevelopmental and temperamental traits that may precede BD onset [43]. Positive family history is the single most reliable predictor of transition to BD, often exceeding the predictive value of individual prodromal symptoms. This is particularly important because many subthreshold features, such as affective lability, irritability, or increased energy, can reflect normative adolescent developmental processes. Risk assessment therefore requires contextual interpretation that integrates family history, symptom persistence, developmental stage, and functional impairment.

Retrospective studies contribute by identifying associations between early symptoms and later emergence of the disorder. Although valuable, these studies rely heavily on the recollections of individuals who have already developed BD, making them susceptible to recall bias, especially when reconstructing early or subclinical experiences. Moreover, when neurobiological correlates are explored retrospectively, it is difficult to distinguish true pre-illness markers from changes resulting from repeated mood episodes, treatment exposure, or illness progression [44].

In contrast, prospective studies—particularly those that follow individuals from the onset of their first manic episode—are currently considered the most informative. These studies avoid many confounds inherent in retrospective designs, such as recall errors and treatment effects, by observing patients in real time. As such, they offer a clearer view of the natural course of BD, including the clinical, cognitive, and functional correlates that may predict long-term outcomes [45,46]. Furthermore, prospective studies can aid in stratifying patients according to their stage of illness, allowing for the development of stage-specific interventions and potentially improving long-term prognosis [47].

However, prospective studies also face limitations. Despite their methodological strengths, they may lack diagnostic specificity, as individuals identified as “at risk” for BD may go on to develop a range of psychiatric disorders, not exclusively BD. Additionally, these studies often struggle with statistical power due to the relatively low incidence of BD in the general population, which can affect the generalizability of their findings [44].

Translating neurobiological insights into clinical practice requires evidence-based intervention strategies tailored to the early phases of illness. When it comes to neurobiological findings, the literature remains inconclusive. Although no single structural or functional neuroimaging marker has emerged as a reliable indicator of genetic liability for BD, recent research points to promising directions. Neuroimaging studies in youth with BD or those at high risk [48,49] have highlighted alterations in brain structure and function, which may reflect underlying neurodevelopmental abnormalities. Notably, a recent meta-analysis suggested that gray matter loss may be a state-related marker of BD, rather than a purely trait-related or genetic one [49]. This finding opens the possibility that structural imaging could help distinguish individuals who are likely to transition to BD from those who will not, particularly when combined with clinical and cognitive data [49].

Emerging technologies and novel analytic techniques hold promise for the identification of biomarkers of disease onset and progression. These include imaging-based endophenotypes that may predict treatment response and support more personalized interventions. However, challenges remain, especially regarding methodological variability, the influence of psychotropic medications, and the heterogeneity of clinical phenotypes. For instance, many first-episode patients in these studies are already undergoing polypharmacotherapy, and the potential neurobiological effects or triggering impact of these treatments are often not accounted for [50,51,52].

In the realm of clinical assessment, progress is being made toward more reliable measurement tools. One example is the Bipolar Prodrome Symptom Interview and Scale-Prospective (BPSS-P), a structured instrument recently validated to assess subthreshold symptoms and risk factors for BD in youth. Tools like the BPSS-P may play a crucial role in the early identification of at-risk individuals, provided they demonstrate sufficient predictive validity and are supported by robust psychometric properties [3]. While the existing literature supports the concept of a prolonged prodromal phase in BD [1,2], a definitive neurobiological or clinical signature of transition remains elusive. Future research will benefit from the integration of multimodal data (genetic, neuroimaging, cognitive, and clinical) within large, longitudinal cohorts. This integrative approach may ultimately enable the identification of clinically meaningful subgroups, improve early detection, and pave the way for preventive interventions tailored to individual risk profiles. Beyond clinical observation, understanding the neurobiological underpinnings of early-stage BD is crucial to identifying objective risk markers and informing targeted intervention.

## 5. Neurobiology of BD

Although neurobiology has not been the central focus of most studies on early intervention in BD, a growing number of investigations have begun to explore the potential role of neurobiological markers, particularly those associated with neuroinflammation, structural and functional brain changes, and connectivity abnormalities, in the pathophysiology and progression of the disorder. Understanding the neurobiological underpinnings of BD, particularly during the prodromal or early stages, could offer essential insights for early detection, prognosis, and the development of targeted treatment strategies. Therefore, neurobiology should be considered an integral component of comprehensive BD management.

To enhance clinical relevance, neurobiological findings can be conceptualized as either trait markers, which reflect stable vulnerability or genetic loading, or state markers, which reflect episode-related changes. Trait markers include structural alterations observed in unaffected first-degree relatives (e.g., subtle reductions in prefrontal cortex thickness, white matter microstructural differences) as well as neurocognitive endophenotypes consistently linked to familial risk. In contrast, state markers encompass dynamic abnormalities such as reduced hippocampal volume, heightened amygdala reactivity, and alterations in fronto-limbic functional connectivity that appear during acute mood episodes and often normalize with remission or treatment. Distinguishing between these two classes of markers is essential for identifying early vulnerability pathways versus neuroprogressive changes emerging with illness onset.

A variety of neuroimaging techniques, including computed tomography (CT), magnetic resonance imaging (MRI), and positron emission tomography (PET), have been employed to investigate brain alterations associated with BD across different stages of the illness [53,54]. While these tools have yielded numerous intriguing results, a persistent challenge remains the identification of robust and specific biomarkers that reliably distinguish individuals with BD or those at high risk from healthy controls or individuals with other psychiatric conditions [55,56]. One of the main difficulties in interpreting neuroimaging data in BD is the considerable heterogeneity across studies, which stems from multiple factors including variations in sample characteristics (e.g., age, illness stage), medication status, and psychiatric or medical comorbidities [57,58]. For instance, the use of mood stabilizers or antipsychotics, often initiated before neuroimaging is conducted, can influence brain structure and functional connectivity, thereby confounding efforts to isolate disorder-specific abnormalities [59,60]. Furthermore, methodological discrepancies in image acquisition, preprocessing, and analysis pipelines contribute to inconsistencies and limit reproducibility across studies [61,62]. These challenges highlight the importance of harmonized, multi-center research protocols and the inclusion of medication-naïve cohorts in future studies to enhance reliability and comparability of findings [63,64]. One of the key aims in this area of research is to identify brain-based abnormalities that could confer vulnerability to BD, serve as protective factors, or reflect early disease processes. Such markers could inform risk prediction models, facilitate earlier diagnosis, and guide more individualized therapeutic approaches. In particular, neuroimaging studies in individuals at genetic risk, such as unaffected first-degree relatives of BD patients, have revealed subtle brain alterations that may correlate with mild neurocognitive impairments, especially in domains such as executive functioning and working memory [65,66,67,68].

Despite a large and expanding body of neurobiological research, findings remain inconsistent, and no single structural or functional imaging marker has emerged as a definitive indicator of genetic liability to BD. Nevertheless, this line of inquiry continues to hold promise. Studies of young patients in early stages of BD have highlighted the potential of neuroimaging to clarify the neurodevelopmental trajectory of the disorder. Such investigations may also help distinguish state-related (episode-dependent) from trait-related (enduring or genetically linked) brain abnormalities [65,68].

Recent advances in imaging protocols, machine learning, and computational neuroanatomy have enabled researchers to begin addressing key clinical questions more precisely, including the potential to identify treatment-relevant endophenotypes. These biological subtypes could inform patient stratification strategies and enhance the personalization of treatment planning, which is a long-term goal in the management of BD. For example, a recent study has employed machine learning models that integrate multimodal data, such as polygenic risk scores, structural MRI, and cognitive profiles, to predict which high-risk individuals are likely to develop syndromal BD within two to five years. Specifically, Zhang et al. [69] applied a support vector machine classifier using MRI and clinical features in high-risk youth and achieved over 80% accuracy in distinguishing converters from non-converters. These predictive algorithms, though still under investigation, hold potential for early diagnosis and precision targeting of interventions.

A recent meta-analysis found that gray matter loss may be more reflective of illness state rather than an inherited trait, suggesting its potential utility in identifying individuals who are transitioning from an at-risk mental state to full-threshold BD [49].

Structural imaging studies in adolescents with BD have also pointed to alterations in white matter connectivity, particularly in neural pathways implicated in emotion regulation, such as fronto-limbic circuits. These alterations may interfere with the development of crucial white matter bundles and related gray matter areas during a critical neurodevelopmental window, thereby contributing to symptom emergence [70]. These findings highlight the need for early intervention before neuropathological changes consolidate. The reduction in hippocampal volume, repeatedly observed in early-stage BD, is thought to reflect impaired neurogenesis and heightened stress responsivity mediated by HPA axis dysregulation [71,72]. This vulnerability may partially explain why early pharmacological interventions such as lithium, known to promote hippocampal neurogenesis and protect white matter integrity, have shown promise not only in mood stabilization but also in potentially reversing or halting neuroprogressive damage [73]. These mechanistic insights support the rationale for using neuroprotective agents in high-risk individuals even before full-blown symptoms emerge.

In addition, volumetric neuroimaging studies have demonstrated that abnormalities in regions such as the prefrontal cortex, amygdala, and hippocampus may precede behavioral symptoms in individuals at risk of BD. Investigating the relationships between these brain changes and genetic susceptibility, illness burden, and treatment exposure, particularly to mood stabilizers like lithium, could enhance our understanding of disease mechanisms and treatment response variability [38].

Neurobiological research in BD represents a rapidly evolving field with substantial potential. Continued efforts to refine imaging methodologies, standardize protocols, and conduct large-scale longitudinal studies are essential to fully harness the promise of neurobiology. The clinical utility of biomarkers such as hippocampal volume or white matter microstructure remains limited in real-world psychiatric settings. These measures are costly, technically demanding, and not routinely available in many clinical environments. Therefore, a more feasible approach may involve integrating such biomarkers with validated clinical instruments like the BPSS-P scale. For instance, combining structural imaging data with prodromal symptom ratings could improve the accuracy of risk stratification models and enhance early diagnostic decision-making, especially in specialized early intervention clinics.

Table 3 summarizes the main replicated neuroimaging findings in early-stage BD, along with their strength of evidence and key references. Translating neurobiological insights into clinical practice requires evidence-based intervention strategies tailored to the early phases of illness. To facilitate synthesis, we rated the consistency of the evidence for each identified prodromal factor or clinical marker. This rating was based on the number of supporting studies, methodological rigor (e.g., longitudinal design, sample size), and the degree of concordance across settings and populations. Ratings were categorized as “high” or “low”.

To improve clinical interpretability, neurobiological findings were reorganized into two categories: (1) trait markers, reflecting stable vulnerability and often detected in asymptomatic high-risk individuals, and (2) state markers, which represent dynamic changes associated with acute mood episodes or neuroprogression. Table 4 summarizes these distinctions, highlighting their clinical relevance and the supporting evidence.

## 6. Early Psychosocial and Pharmacological Interventions

Early intervention in BD, particularly during the prodromal phase or following a first manic episode, represents a crucial window to influence the course of illness and reduce long-term disability [74,75]. Intervening at these early stages can potentially prevent full syndromal onset [76], delay relapse [77], and limit the cumulative burden of mood episodes, including their impact on cognitive and functional outcomes [78,79]. The clinical staging model has offered a valuable framework for determining the timing and intensity of interventions, proposing that less intensive, low-risk strategies, such as psychoeducation or psychotherapy, may be particularly suitable in early or subthreshold stages [77], whereas pharmacological treatment becomes more appropriate as symptom severity and diagnostic certainty increase [80].

### 6.1. Psychosocial Interventions

Comparative studies evaluating psychosocial interventions have shown variable efficacy depending on illness stage. Cognitive-behavioral therapy (CBT) appears most effective in subthreshold or early-stage patients, particularly for managing depressive symptoms and promoting insight [30,81]. Family-focused therapy (FFT) demonstrates stronger benefits in youth with symptomatic BD and familial loading, notably in delaying relapse and improving mood regulation [82]. Interpersonal and social rhythm therapy (IPSRT) shows promise across stages, particularly when circadian rhythm disruption is prominent [83]. Meta-analyses suggest FFT may yield the largest effect sizes on recurrence prevention in adolescents, whereas CBT is more effective in reducing residual symptoms [84]. Each psychosocial intervention targets specific underlying mechanisms. CBT enhances cognitive control and restructuring of maladaptive thought patterns, thereby improving mood regulation. FFT focuses on psychoeducation, emotional communication, and conflict resolution within the family system, reducing expressed emotion and relapse risk. IPSRT aims to stabilize circadian rhythms through regular daily routines and interpersonal role clarity, addressing the heightened sensitivity to sleep–wake disruptions commonly seen in BD. These mechanism-based approaches align with the neurodevelopmental vulnerabilities identified in early-stage BD [28,29].

### 6.2. Pharmacological Interventions

From a pharmacological perspective, interventions in early-stage BD remain a subject of ongoing debate. The use of lithium, long considered the gold standard mood stabilizer, has garnered interest not only for its mood-stabilizing effects but also for its neuroprotective properties, including the preservation of hippocampal volume and white matter integrity [49,85]. Early lithium treatment may mitigate neuroprogressive changes and preserve cognitive functioning, particularly executive function and working memory, which are often compromised early in the illness [85]. Low-dose atypical antipsychotics, such as quetiapine or aripiprazole, have also been explored in first-episode mania, with some evidence of efficacy and tolerability, although concerns about metabolic side effects and long-term use persist [86]. Atypical antipsychotics may offer mood stabilization but carry a significant risk of metabolic syndrome, especially with prolonged use in adolescents. These risks must be carefully weighed against clinical benefits, and early-stage patients may benefit more from low-intensity or non-pharmacological options until clear diagnostic thresholds are met.

Importantly, early pharmacological intervention must balance potential benefits, such as relapse prevention and symptom control, with the risks of overtreatment, especially in patients with ambiguous symptom profiles or uncertain diagnostic trajectories. As such, current guidelines increasingly advocate for a stepwise, personalized approach informed by familial risk, functional status, symptom severity, and patient preferences. In individuals presenting with subthreshold symptoms and strong familial loading, psychosocial interventions may be initiated first, with pharmacological treatments considered in cases of escalating symptoms or clear syndromal transition [17,87,88,89].

The development of biomarkers, neuroimaging markers, and clinical prediction tools may in the future enhance risk stratification and treatment personalization. However, until these tools become more accurate, accessible, and cost-effective, clinical judgment supported by structured assessment tools remains essential [90,91].

At present, our ability to detect early clinical or biological indicators that reliably predict BD remains limited by heterogeneity across studies and a lack of standardized diagnostic methodologies. Despite these challenges, evidence supports the potential of early intervention strategies, especially during subthreshold and high-risk stages, to alter the illness trajectory and preserve cognitive and psychosocial functioning. Future progress will depend on large-scale, longitudinal, and multimodal studies that integrate clinical, genetic, and neurobiological data to enhance prediction and personalize care. Advancing early identification and intervention in BD will require a shift toward scalable, validated, and stage-specific tools capable of guiding timely and effective preventive strategies. Recent frameworks propose stage-specific strategies incorporating biomarkers, psychosocial risk, and clinical trajectory to guide early personalized interventions [92,93,94]. Key research priorities include exploring gene–environment interactions and their impact on brain maturation, as well as the role of dynamic neuroplasticity in illness progression. Longitudinal studies integrating digital phenotyping (e.g., passive smartphone data, sleep and activity monitoring) with serial neuroimaging and genetic profiling could offer unprecedented insights into early illness trajectories [95,96,97]. For example, the RADAR-CNS project (Remote Assessment of Disease and Relapse in Central Nervous System Disorders) has pioneered the use of wearable sensors and smartphone-based data streams to predict mood instability and relapse risk in affective disorders. Such multimodal approaches may ultimately enable the identification of individualized risk signatures and improve the timing and precision of preventive interventions [98].

## 7. Discussion and Limitations

Synthesizing evidence across clinical, developmental, and neurobiological domains suggests a convergent model in which early risk for BD emerges from the interaction of inherited vulnerability, neurodevelopmental processes, and evolving clinical features. Familial-genetic loading appears to shape early temperament and stress sensitivity, which in turn modulate emotional reactivity and the maturation of fronto-limbic circuits. Developmental disruptions, such as sleep–wake instability, affective lability, and attentional dysregulation, may represent early phenotypic expressions of these underlying vulnerabilities. Neurobiological studies support this trajectory, indicating that trait-like alterations in prefrontal and limbic structures can predate illness onset, while state-related changes in functional connectivity and subcortical activation emerge closer to the transition to syndromal episodes [2,16].

In this model, early clinical symptoms do not appear in isolation but rather reflect an unfolding interaction between neurodevelopmental maturation, environmental stressors, and neural circuitry dynamics. This integrative perspective highlights the importance of multimodal, longitudinal assessment and underscores why early intervention must target not only symptom reduction but also sleep–wake regulation, stress reactivity, and family environment, domains that bridge biological vulnerability and clinical expression. Such a unified framework provides a foundation for more precise risk stratification and personalized preventive strategies.

Evidence supports the potential of early intervention strategies to alter the trajectory of BD, particularly during the ultra-high-risk (UHR) or Stage I phases, when symptoms are subsyndromal but increasingly specific. Intervening at these stages may prevent progression to syndromal illness and reduce the likelihood of subsequent cognitive decline and functional impairment.

Several recent studies have also proposed the use of biomarkers and advanced neuroimaging techniques to detect BD in its earliest stages. Although promising, these approaches remain limited in clinical practice due to the lack of specific, validated predictive markers [66,67]. Although early detection and intervention strategies for BD show promise, their translation into routine clinical practice is constrained by several health-system barriers. First, access to specialized early-intervention services remains uneven across regions, with many healthcare systems lacking dedicated pathways for youth at risk or for individuals presenting with subthreshold symptoms. Second, provider-level factors, such as limited training in the recognition of prodromal features and insufficient familiarity with staging models, reduce the likelihood that early signs are systematically identified. Third, reimbursement structures often prioritize acute treatment over preventive or developmental approaches, creating disincentives for longitudinal monitoring or family-based interventions. Finally, fragmented service organization and poor coordination between primary care, child–adolescent services, and adult mental health programs hinder continuity of care precisely during the developmental periods in which early bipolar features typically emerge [84]. Addressing these system-level challenges is essential to bridge the translational gap and to support the real-world implementation of evidence-informed early intervention frameworks.

Expanding early detection efforts in BD raises important ethical concerns, particularly regarding the risk of premature labeling and its potential unintended consequences. Identifying an adolescent or young adult as “at risk” may inadvertently lead to stigma, altered self-concept, or increased anxiety in both patients and families. There is also a risk of overdiagnosis or unwarranted pharmacological treatment when early symptoms are nonspecific or developmentally normative. These challenges are compounded by uncertainties in predicting individual illness trajectories, which can result in false positives or unnecessary monitoring.

To mitigate these risks, ethically grounded early intervention models emphasize transparent communication of probabilistic risk, shared decision-making, and stepped-care approaches that prioritize low-risk psychosocial strategies before considering medication. Additionally, periodic reassessment, ongoing psychoeducation, and the integration of family perspectives help ensure that early detection does not become a source of harm. These strategies support the responsible implementation of early identification frameworks while safeguarding patient autonomy and well-being.

The current evidence base for early intervention in BD is heterogeneous, with certain approaches demonstrating consistently stronger support. Among psychosocial interventions, FFT and CBT show the most robust evidence, particularly in youth with familial risk or subthreshold symptoms, with multiple trials demonstrating reductions in mood symptoms, improved functioning, and delayed recurrence [30,81,82]. Although CBT and FFT demonstrate moderate evidence for reducing subthreshold mood symptoms and improving functioning in high-risk youth, current trials do not conclusively show that these treatments prevent transition to BD. Their use in early phases is primarily justified by their favorable safety profile and developmental appropriateness.

IPSRT also benefits from moderate-quality evidence, especially in individuals presenting early disturbances in circadian regulation [83]. In contrast, pharmacological strategies remain more cautiously applied: while lithium has strong evidence as a mood stabilizer and potential neuroprotective agent in syndromal early episodes [49,85], its role in prodromal states remains insufficiently studied. Despite evidence supporting lithium’s neuroprotective properties, its use in prodromal stages is constrained by risks of overtreatment, potential side effects, and the absence of diagnostic certainty. Current guidelines recommend its use primarily after a first syndromal episode, while psychosocial interventions are preferred in earlier, ambiguous phases.

Trials of atypical antipsychotics, omega-3 fatty acids, and anti-inflammatory agents show preliminary or mixed results and should be considered exploratory [86]. Finally, emerging approaches such as digital phenotyping, passive monitoring, and polygenic or multimodal risk algorithms remain experimental and require substantial validation before clinical implementation [90,91]. Distinguishing these levels of evidence is essential to guide clinicians toward interventions with established benefits while avoiding premature adoption of unproven strategies.

While our review highlights several promising early intervention strategies for BD, limitations must be acknowledged regarding their generalizability and real-world application. First, much of the evidence stems from studies conducted in high-income countries with robust healthcare infrastructures, potentially limiting their applicability in low- and middle-income settings. In addition, cultural factors, such as stigma toward mental illness, familial roles, and divergent explanatory models of mood symptoms, may significantly influence help-seeking behavior, early detection, and the acceptability of interventions. Our understanding of the prodromal phase of BD remains insufficient to reliably identify early clinical events that predict disease onset with a high degree of certainty. This limitation is largely attributable to significant heterogeneity across studies, particularly with respect to patient selection criteria, study design, and outcome measures. Such variability hampers the ability to draw generalizable conclusions and undermines efforts to establish early diagnostic or prognostic indicators. Importantly, most prodromal symptoms remain nonspecific and frequently overlap with normative adolescent variability and other psychiatric conditions. Similarly, neuroimaging findings, although informative at a group level, lack the specificity required for individual prediction. These limitations reinforce the need for multimodal risk stratification and longitudinal assessment rather than reliance on single clinical or biological indicators.

Given the current state of knowledge, the development and implementation of standardized diagnostic methodologies for the screening and evaluation of early symptoms would be of great value. A major limitation in the characterization of early symptom development in BD is the reliance on retrospective self-report or parent-report. Retrospective accounts are vulnerable to multiple sources of bias: individuals may reinterpret earlier experiences through the lens of a confirmed diagnosis, parents may under- or over-estimate early emotional or behavioral disturbances, and both may be influenced by mood-congruent memory distortions. These biases are particularly problematic in BD, where fluctuations in mood state can directly affect recall accuracy. Moreover, retrospective methods tend to blur the temporal boundaries between normative developmental variability and clinically meaningful prodromal features. As a result, the apparent presence, timing, and sequence of early symptoms may be distorted, limiting the reliability of inferences drawn from retrospective data. This reinforces the need for prospective, longitudinal approaches that incorporate structured assessments, ecological momentary monitoring, and objective measures such as sleep–wake rhythms or cognitive performance to more accurately capture the evolution of early bipolar features.

## 8. Conclusions

Early warning signs and prodromal symptoms of BD are often subtle and overlap with normative adolescent experiences, particularly in cultures where emotional expression is discouraged or pathologized differently. This may lead to underdiagnosis or misinterpretation of emerging mood dysregulation, especially among ethnic minorities or individuals with limited access to mental health literacy [99].

In terms of implementation, even in systems with structured mental health services, barriers remain. These include limited availability of trained clinicians, lack of integration between primary care and psychiatric services, and challenges in sustaining longitudinal monitoring and psychoeducation for at-risk individuals. Digital tools and community-based screening could partially address these gaps, but they require rigorous validation and culturally tailored adaptations before widespread deployment.

Moreover, ethical concerns surrounding early intervention, such as potential labeling, overtreatment, or medicalization of distress, must be carefully navigated, particularly when intervening in youth or in subsyndromal phases. Programs must prioritize a trauma-informed, person-centered approach that values autonomy and minimizes harm. Despite existing challenges, early detection and intervention may reduce long-term morbidity in BD. Continued investment in longitudinal studies, standardized assessment tools, and multimodal research strategies, including neurobiological and genetic investigations, will be essential in advancing the field toward more precise and personalized care for individuals at risk of BD. Advancing early identification and intervention in BD will require a shift toward large-scale, multimodal, and longitudinal studies that integrate clinical, cognitive, and biological data to inform stage-specific and personalized preventive strategies.

## Figures and Tables

**Table 1:** Prodromal Symptoms and Interventions in Bipolar Disorder (BD).

Prodromal Symptom	Typical Age of Onset	Predictive Value for BD Onset	Potential Clinical Interventions	References
Mood lability/emotional instability	Adolescence to early adulthood	Moderate to high (PPV: 30–50%)	Psychoeducation, emotional regulation strategies, family therapy	Cannon et al., 2016 [28]; Tandon et al., 2012 [29]
Subclinical depressive symptoms	Adolescence	Moderate	CBT, mood monitoring	Weintraub et al., 2022 [30]; Van Meter et al., 2016 [31]
Cyclothymic features	Childhood to adolescence	Moderate	Psychoeducation, temperament-focused therapy	Duffy et al., 2010 [32]; Hafeman et al., 2016 [33]
Irritability	Childhood to adolescence	Moderate	Anger management strategies, CBT, family support	Faedda et al., 2014 [34]; Duffy et al., 2009 [35]
Racing thoughts	Adolescence	Moderate	CBT, mindfulness-based approaches	Leopold et al., 2012 [36]; Van Meter et al., 2016 [31]
Increased psychomotor activity	Adolescence	Moderate	Behavioral activation, physical activity programs	Martini et al., 2021 [37]; Duffy et al., 2009 [32]
Anxiety	Adolescence	Low to moderate	CBT, anxiety management techniques	Van Meter et al., 2016 [31]; Sala et al., 2020 [38]
Sleep disturbances	Early childhood to adolescence	Low	Sleep hygiene education, behavioral interventions	Ng et al., 2015 [39]; Salvatore et al., 2008 [40]
Excessive crying (early childhood)	Toddlerhood (2–4 years)	Low (nonspecific)	Parental guidance, early developmental monitoring	Luby et al., 2006 [41]; Birmaher et al., 2009 [42]

Abbreviations. CBT: Cognitive Behavioral Therapy; PPV: Positive Predictive Value.

**Table 2:** Prodromal Markers with Strongest Predictive Validity for Transition to Bipolar Disorder (BD).

Prodromal Marker	Predictive Validity	Key Findings/Direction	References
Subthreshold hypomanic symptoms	High	Strong predictor; 3–5× higher transition risk when persistent	Martini et al. [37]; Duffy et al. [32]
Affective lability/mood swings	High	Episodic mood instability predicts BD onset, especially with family history	Cannon et al. [28]; Tandon et al. [29]
Cyclothymic/irritable temperament	Moderate–High	Stable temperament traits increasing long-term BD risk	Duffy et al. [32]; Hafeman et al. [33]
Sleep–wake dysregulation	Moderate	Circadian variability and reduced sleep duration precede mood episodes	Ng et al. [39]; Salvatore et al. [40]
Psychotic-like experiences	Moderate	Increased BD risk when co-occurring with mood dysregulation	Van Meter et al. [31]; Faedda et al. [34]
Anxiety symptoms	Low–Moderate	General risk factor but low specificity for BD	Van Meter et al. [31]; Sala et al. [38]
Racing thoughts	Moderate	Often precedes hypomanic episodes in high-risk youth	Leopold et al. [36]; Van Meter et al. [31]
Psychomotor activation	Moderate	Increased motor activity commonly reported pre-onset	Martini et al. [37]; Duffy et al. [32]
Early childhood signs (crying, sleep dysregulation)	Low (nonspecific)	Nonspecific neurodevelopmental vulnerability markers	Luby et al. [41]; Birmaher et al. [42]

**Table 3:** Key Neuroimaging Findings in Early-Stage Bipolar Disorder.

Brain Region	Alteration Type	Effect Direction	Typical Sample Sizes	Evidence Consistency	References
Hippocampus	Volume reduction	↓ hippocampal volume	*n* ≈ 40–150	High	Hajek et al. [70]
Amygdala	Mixed volumetric findings	↑/↓ depending on mood state	*n* ≈ 30–120	Moderate	Fusar-Poli et al. [49]
Prefrontal cortex	Reduced thickness/connectivity	↓ cortical thickness; ↓ connectivity	*n* ≈ 50–200	High	Duffy et al. [17]
Anterior cingulate cortex	Functional hypoactivation	↓ activation during emotion tasks	*n* ≈ 20–80	Moderate	Keener & Phillips [68]
White matter tracts	Reduced integrity (DTI)	↓ FA; ↑ MD	*n* ≈ 40–160	Moderate–High	Hajek et al. [70]

Note: The level of “evidence consistency” was assessed based on the number of independent studies reporting similar findings, the methodological quality and design of the studies (e.g., prospective vs. retrospective), and the degree of convergence across different populations and settings. “High consistency” refers to findings replicated in multiple high-quality studies with low methodological heterogeneity, while “low consistency” indicates limited replication, divergent methodologies, or inconsistent results across studies. Abbreviations: FA, fractional anisotropy; MD, mean diffusivity; DTI, diffusion tensor imaging; ↓, decrease; ↑, increase.

**Table 4:** Trait vs. State Neurobiological Markers in Bipolar Disorder.

Category	Definition	Representative Findings	Clinical Implications	References
Trait markers	Stable neurobiological characteristics reflecting genetic vulnerability or enduring risk, observed even in asymptomatic or unaffected high-risk individuals.	Subtle reductions in prefrontal cortical thickness White matter microstructural abnormalities in first-degree relatives Executive function and working memory deficits preceding onset Altered fronto-limbic connectivity patterns in high-risk youth	Useful for risk stratification Indicate neurodevelopmental vulnerability Potential targets for early prevention models	Duffy et al. [32]; Hafeman et al. [33]; Phillips et al. [58]; Piguet et al. [62]
State markers	Dynamic, episode-dependent neurobiological changes fluctuating with mood state, illness severity, or treatment exposure.	Reduced hippocampal volume in acute illness Amygdala hyperactivation during manic/depressive episodes Anterior cingulate functional dysconnectivity during episodes Gray matter reductions linked to neuroprogression	Reflect illness activity Useful for monitoring treatment response Provide insight into neuroprogressive mechanisms	Fusar-Poli et al. [49]; Salvatore et al. [40]; Hajek et al. [70]; Keener & Phillips [68]

Note. Trait markers refer to stable neurobiological characteristics associated with genetic vulnerability or enduring risk, typically observable in asymptomatic first-degree relatives or high-risk individuals. These markers tend to remain consistent across time and reflect long-term susceptibility rather than acute illness expression. State markers refer to dynamic neurobiological alterations that fluctuate according to current mood state, episode severity, or treatment exposure. They often normalize during remission and are therefore considered indicators of illness activity rather than vulnerability. The classification presented in this table is based on the convergence of longitudinal, neuroimaging, and endophenotype studies, as well as replication across independent samples.

## Data Availability

No new data was created for this study.
